# Clinical characteristics and mortality predictors of patients with cancer hospitalized by COVID-19 in a pediatric third-level referral center

**DOI:** 10.3389/fped.2022.960334

**Published:** 2022-07-28

**Authors:** Laura-Monserrat Hernández-Regino, Manuel De Jesús Castillejos-López, Arnoldo Aquino-Gálvez, Liliana Velasco-Hidalgo, Alda García-Guzmán, Marco Aguilar-Ortiz, Rocío Cárdenas-Cardos, Luz María Torres-Espíndola

**Affiliations:** ^1^Laboratory of Pharmacology, National Institute of Pediatrics, Mexico City, Mexico; ^2^Department of Hospital Epidemiology and Infectology, National Institute of Respiratory Diseases “Ismael Cosío Villegas,” Mexico City, Mexico; ^3^Laboratory of Molecular Biology, Pulmonary Fibrosis Department, National Institute of Respiratory Diseases “Ismael Cosío Villegas,” Mexico City, Mexico; ^4^Oncology Service, National Institute of Pediatrics, Mexico City, Mexico

**Keywords:** COVID-19, cancer, pediatrics, mortality, predictor

## Abstract

**Background:**

More than 135 million COVID-19 cases (coronavirus disease 2019) have been reported worldwide until today, with over 2.9 million deaths. Several studies have demonstrated that disease severity is lower in the pediatric population than in adults; however, differences are described in patients with chronic diseases, including oncological patients. Current world literature suggests patients with comorbidities, including cancer, have an increased risk of unfortunate outcomes. Therefore, our objective was to describe the clinical characteristics and epidemiological factors associated with mortality in a cohort of pediatric cancer patients hospitalized for COVID-19.

**Methods:**

This is a retrospective, descriptive study of the cases of patients with cancer hospitalized for COVID-19. A total of 40 pediatrics were included in the analysis. Data from pediatric patients with COVID-19 included clinical and epidemiological records, laboratory, imaging studies, COVID-19 diagnostic methods, and medical treatment.

**Results:**

Of the 40 pediatric patients admitted with cancer with a confirmed diagnosis of COVID-19, 42.5% were solid tumors, 40% leukemias, and 17.5% lymphomas. The clinical parameters associated with mortality were stage IV tumor (*p* = 0.029) and intubation (*p* < 0.001). The biochemical factors associated with lower survival were thrombocytopenia under 25,000 cells/mm^3^ (*p* < 0.001), D-dimer over 1 μg/ml (*p* = 0.003), clinical malnutrition (*p* = 0.023), and disseminated intravascular coagulation (*p* = 0.03).

**Conclusion:**

Our findings showed that the fever was the most frequent symptom, and the clinical parameters associated with mortality were stage IV tumor, intubation, saturation percentage, RDW, platelets, creatinine, ALT, D-dimer, ferritin, and FiO2 percentage. The thrombocytopenia, D-dimer, nutritional status, and disseminated intravascular coagulation were significantly associated with lower survival.

## Introduction

Cancer is the second leading cause of death among children between 1 and 14 years, only after accidents (1). Children with cancer undergoing treatment are vulnerable populations since treatment causes immunosuppression increasing the probability of contracting infections with the highest severity (2).

Coronavirus disease 2019 incidence in children with cancer has been previously reported to be higher than general pediatric population (3). The symptoms severity in children with cancer affected by COVID-19 has also been reported to be higher than general pediatric population, with greater odds of developing severe symptoms and the need for oxygen therapy (1).

Children with cancer are a high-risk population (4); however, the specific data concerning COVID-19 results in the oncologic pediatric population are limited, and there is a lack of extensive testing based on the population regarding the risk for children with cancer as well. Therefore, the need for mortality prognostic tools in COVID-19-infected patients from the Mexican children population arises. Therefore, our objective was to describe the clinical characteristics and epidemiological factors associated with mortality in a cohort of pediatric cancer patients hospitalized for COVID-19.

## Materials and methods

A retrospective cohort study was conducted on pediatric patients with cancer receiving attention at the Oncology service of the National Institute of Pediatrics in Mexico City, who were hospitalized due to COVID-19 compatible symptoms and were undergoing oncological treatment from March 2020 to 2021; the collected data included clinical and epidemiological records, laboratory, imaging studies, COVID-19 diagnostic method, and medical treatment; follow-up for patients was realized on admission and during hospitalization.

Because it is a retrospective study, all data were gathered from patients‘ electronic records through a standard collection data form.

### Statistical analysis

The frequency of symptoms, medians, and interquartile ranges (Q_25_-Q_75_) was analyzed for the quantitative variables; Kaplan–Meier curves were made using the log-Rank test to evaluate the COVID-19 mortality-associated factors. Values of *p* < 0.05 were considered statistically significant. The SPSS (Statistical Package for the Social Sciences) program version 21.0 (IBM Corp., Armonk, New York, United States) was used for all statistical tests.

Categorical variables were described as numbers (percentage), quantitative variables as mean (standard deviation) if data had a normal distribution, and median and interquartile range (Q_25_-Q_75_) if they had a non-normal distribution. The Chi-square test was used to evaluate the statistical significance of the dichotomous variables and the Mann–Whitney U test for quantitative variables. A *p* < 0.05 value was considered statistically significant.

The survival probability of the different subgroups was the primary outcome studied through the Kaplan–Meier (KM) graphic using comorbidity, symptoms, and cancer-type subgroups. Log-Rank (Mantel–Cox) tests were used to measure survival curve differences.

A non-probabilistic sample of consecutive cases was obtained, which were included if they correctly met the selection criteria previously described.

## Results

### Demographic features of the study population

A total of 40 hospitalized COVID-19 patients from 1 March to 30 September 2021, met the selection criteria. The diagnosis was solid tumors in 17 (42.5%), acute leukemia in 16 (40%), and lymphomas in 7 (17.5%). The age median was 11 years (Q_25_5.25-Q_75_14); 27 (71%) were male patients and 13 (32.5%) female patients. The solid tumors of the central nervous system (CNS) were the most frequent, 3 (7.5%). Patients in the advanced stage were 12 (71%). Malnutrition was the most common comorbidity, 6 (15%), followed by hypothyroidism (5%) and anemia 2 (5%). Of the 40 patients, all were on active treatment (within 14 days before the onset of symptoms); of which 35 patients received chemotherapy, 2 were with radiotherapy, and 3 were post-surgery patients (Table 1).

**TABLE 1:** General characteristics of the cohort.

Characteristic	Frequency (%)
**Type of Cancer**	
Solid tumor	17(42.5)
Leukemia	16(40)
Lymphoma	7(17.5)
Median age (years)	11
**Sex**	
Female	13(32.5)
Male	27(67.5)
**Type of solid tumor**	
CNS tumors	3(7.5)
Retinoblastoma	3(7.5)
Osteosarcoma	2(5)
Neuroblastoma	2(5)
Hepatic	2(5)
Histiocytosis	2(5)
Germinal tumor	1(2.5)
Ewing Sarcoma	1(2.5)
Wilms tumor	1(2.5)
** Stage (solid tumors *N* = 17)**	
Advanced	12(71)
Localized	5(29)
**Risk (leukemias *N* = 16)**	
High	12(75)
Standard	4(25)
**Treatment stage**	
Chemotherapy	35(87.5)
Radiotherapy	2(5)
Post-surgical	3(7.5)
**Comorbidities**	
Malnutrition	6(15)
Hypothyroidism	2(5)
Anemia	2(5)
Insulin resistance	1(2.5)
Without comorbidity	29(72.5)

### Clinical manifestations in cancer and COVID-19 pediatric patients

The most frequent symptoms at admission encompass fever *N* = 32 (80%), tachycardia *N* = 20 (50%), and cough *N* = 14 (35%), Table 2. All patients applied a test to detect the presence of the virus that causes COVID-19, and in 11 patients, the test performed was by antigen detection and in 29 by polymerase chain reaction, finding that in *N* = 40 (100%), the result was positive; the hypoxia evaluation showed that *N* = 28 (70%) had mild, *N* = 9 (22.5%) had severe, and *N* = 3 (7.5%) had moderate hypoxia. Regarding treatment, *N* = 29 (72%) patients were given paracetamol and *N* = 9 (22.5%) dexamethasone.

**TABLE 2:** Frequency of clinical manifestations in children with cancer and COVID-19.

Symptom	Frequency (%)
Fever	32 (80)
Tachycardia	20 (50)
Cough	14 (35)
Sudden onset	10 (25)
Rhinorrhea	10 (25)
Cephalea	8 (20)
Arthralgia	7 (17.5)
Asthenia	7 (17.5)
Odynophagia	7 (17.5)
Adynamia	6 (15)
Attack on the general status	6 (15)
Irritability	6 (15)
Dyspnea	5 (12.5)
Lung rattle	5 (12.5)
Myalgia	5 (12.5)
Nasal congestion	4 (10)
Abdominal pain	4 (10)
Chills	4 (10)
Altered breathing	4 (10)
Conjunctivitis	3 (7.5)
Lymphadenopathy	3 (7.5)
Diarrhea	2 (5)
Thoracic pain	2 (5)
Vomit	2 (5)
Alteration in the bite	1 (2.5)
Anosmia	1 (2.5)
Anxiety	1 (2.5)
Dysgeusia	1 (2.5)
Expectoration	1 (2.5)

### Most frequent complications in cancer and COVID-19 pediatric patients

Coronavirus disease 2019-derived complications in this cohort were acute respiratory failure in *N* = 30 (75%), cases that required invasive mechanical ventilation (IMV) in *N* = 8 (20%), added infections in *N* = 9 (22.5%), pediatric multisystem syndrome in *N* = 7 (17.5%), and acute kidney injury in *N* = 6 (15%) with progression to septic shock in *N* = 5 (12.5%). The calculated median for hospitalization from the beginning of symptoms until discharge was 7.5 days (Q_25_3-Q_75_14), and *N* = 6 (15%) documented deaths were associated with COVID-19 (Table 3).

**TABLE 3:** Frequency of COVID-19 associated complications in cancer pediatric patients.

Variable	Frequency (%)
Acute respiratory failure	30 (75)
Added infections	9 (22.5)
Pediatric multisystem inflammatory syndrome	7 (17.5)
Acute kidney injury	6 (15)
Metabolic acidosis	6 (15)
Pleural effusion	5 (12.5)
Septic shock	5 (12.5)
Cardiac failure	4 (10)
Hydro-electrolytic disorder	4 (10)
Metabolic alkalosis	4 (10)
Disseminated intravascular coagulation	3 (7.5)
Respiratory acidosis	3 (7.5)
Kidney failure	2 (5)
Respiratory alkalosis	2 (5)
Atelectasis	1 (2.5)
Hemodialysis	1 (2.5)
Bronchiectasis	0 (0)

### Comparison of clinical factors among dead and alive associated with mortality in cancer and COVID-19 pediatric patients

From the 40 patients analyzed, *N* = 6 (15%) patients died. The bivariate analysis revealed that all deceased patients had the advanced oncological disease (stage IV) and were undergoing treatment at the moment of the infection (*p* = 0.029); it was also found that *N* = 5 (83.3%) deceased patients were intubated patients (*p* = 0.001), Table 4. Other biochemical and clinical variables were evaluated, such as pre-hospitalization days (days from the beginning of symptoms to hospitalization), intubation days, respiratory rate, blood pressure, temperature, saturation, and hematologic parameters. Findings in deceased patients were a saturation percentage median of 82 (Q_25_ 70.2-Q_75_89) *p* = 0.002, red cell distribution width (RDW) percentage median of 15.3 (Q_25_12.9-Q_75_15.8) *p* = 0.016, platelet count median of 13,500 (Q_25_10750-Q_75_9300) *p* = 0.029, creatinine median of 0.59 (Q_25_0.49-Q_75_1.46) *p* = 0.029, GPT/ALT median of 20 (Q_25_16-Q_75_34.5) *p* = 0.037, D-dimer median of 3.17 (Q_25_0.73-Q_75_6.7) *p* = 0.015, ferritin median of 7,543 (Q_25_2108.5-Q_75_54700) *p* = 0.047, and a FiO_2_ median of 77.5 (Q_25_27.75-Q_75_100) *p* = 0.02; all these factors were associated with mortality in this cohort of patients, Table 5.

**TABLE 4:** Mortality-associated clinical factors comparison.

Variable	Deceased *N* = 6 (%)	Alive *N* = 30 (%)	*p*
Male	66.7	66.7	1
Acute leukemia	33.3	43.3	0.307
Lymphoma	16.7	13.3	0.307
Osteosarcoma	16.7	3.3	0.307
Germinal tumor	16.7	0	0.307
Histiocytosis	16.7	3.3	0.307
Stage IV	100	33.3	0.029>*
High risk	100	70	0.303
Chemotherapy	100	76.7	0.784
Intubation	83.3	10	0.001>*
Outpatient	66.7	76.7	0.627
Previous treatment	83.3	50	0.196
Fever	100	76.7	0.317
Tachycardia	83.3	46.7	0.182
Cough	50	30	0.378
Myalgia	0	16.7	0.564
Anosmia	16.7	0	0.167
Rhinorrhea	16.7	30	0.655
Dyspnea	16.7	6.7	0.431
Lung rattle	33.3	10	0.186

**TABLE 5:** Clinical and biochemical factors associated with mortality in oncological pediatric patients with COVID-19.

Variable	Deceased (*N* = 6)			Alive (*N* = 30)			*p*
	Median	Q_25_	Q_75_	Median	Q_25_	Q_75_	
Age (years)	12.5	9.75	15.25	9	4	14	0.159
Days before admission	0.5	0	1.75	1	1	2	0.217
Intubation days	5	2	9.5	12	6		0.143
Hospital stay days	13	4	18.5	7	3	14	0.371
Weight(kg)	34.5	26	42.25	23	13	42.25	0.394
Height(cm)	145	123.5	152.8	123	95	149.25	0.172
BMI (kg/m^2)^	16.81	15.92	18.79	17.74	15.97	21.31	0.605
Respiratory rate (bpm)	25	21	35	22	18.75	32	0.576
Heart rate (bpm)	127.5	94.75	144.3	105	89.5	142.25	0.52
Blood pressure mean (mmHg)	71.5	55.75	84.5	76.66	70	88	0.307
Temperature (°C)	37.95	37.72	38.4	36.95	36.47	38.07	0.078
Saturation (%)	82	70.25	89	94	90.75	97.25	0.002*
Leukocytes (cell/mm^3)^	2100	100	7025	4000	2450	7500	0.201
Neutrophils (cell/mm^3^)	850	0	3375	2300	925	5525	0.135
Lymphocytes (cell/mm^3^)	250	0	1725	1000	675	2225	0.103
Monocytes (cell/mm^3^)	150	0	700	600	275	700	0.159
Hemoglobin (g/dL)	9.25	8.1	10.32	10.3	9.9	10.7	0.113
Hematocrit (%)	26.4	23.15	30.4	29.8	28.9	31.9	0.086
MCV (fL)	87.1	86.4	88.8	90.35	87	92.6	0.105
MCH (q/dL)	32.8	29.95	48.3	31.3	29.95	32.3	0.418
RDW (%)	15.35	12.97	15.8	18.15	15.2	20.47	0.016*
Platelets (cell/mm^3^)	13500	10750	9300	252000	40250	306250	0.029*
Glucose (mg/dL)	127	76	255.5	103	84.25	120	0.299
Creatinine (mg/dL)	0.59	0.49	1.46	0.39	0.28	0.58	0.029*
Urea (mg/dL)	24	12.3	76.15	23	11.85	34.6	1
BUN (mg/dL)	7.3	4.9	35.6	9.2	5.5	13.6	0.789
Total bilirubin (mg/dL)	0.42	0.365	1.185	0.69	0.46	0.91	0.275
Direct bilirubin (mg/dL)	0.12	0.075	0.525	0.12	0.8	0.55	0.787
Indirect bilirubin (mg/dL)	0.37	0.255	0.66	0.43	0.37	0.77	0.385
GOT/AST (UI/L)	20	14	85.5	38	24	51.5	0.169
GPT/ALT (UI/L)	20	16	34.5	42	24.5	68.5	0.037*
LDH (UI/L)	382	139	5954	236	158.5	302	0.448
ALP (UI/L)	331	132	444	161	119.25	274	0.201
Sodium (mmol/L)	136.5	34	140.8	137	136	140	0.881
Potassium (mmol/L)	3.75	3.27	4.32	4.2	3.5	5.2	0.685
Chlorine (mmol/L)	103	96.75	110.5	106	104	109	0.593
Phosphorous (mg/dl)	3.95	3.37	5.05	4.2	3.5	5.2	0.685
Magnesium (mg/dl)	1.95	1.75	3.87	2	1.8	2.2	0.949
Calcium (mg/dl)	8.5	8.25	8.82	8.9	8.4	9.4	0.218
PT (s)	14.2	12.15	16.82	12.9	11.9	14.2	0.312
aPTT (s)	33.8	30.85	35.85	33.9	29.1	35.6	0.949
INR	1.26	1.04	1.47	1.12	1.03	1.21	0.334
C-reactive protein (mg/dl)	11.1	5.05	19.27	6	0.9	15.97	0.367
D-dimer (ug/mL)	3.17	0.73	6.7	0.37	0.27	0.89	0.015*
Ferritin (ng/mL)	7543	2108.5	54700	853	217.25	3815	0.047*
Fibrinogen (mg/dl)	536	292.5	623.5	393	288	536	0.367
Procalcitonin (ng/mL)	1.85	0.545	28.31	0.86	0.34	1.41	0.265
pH_1	7.32	7.2	7.42	7.43	7.31	7.49	0.074
pCO_2__7 (mmHg)	45.2	35.25	50.25	31.7	27.6	46	0.16
pCO_2__14 (mmHg)	33	22.05	45.55	37.1	31.3	41.4	0.595
FiO_2_ (%)	77.5	27.75	100	21	21	31	0.02*
PEEP (cmH_2_O)	4.5	1	8.75	5	0	6	1
PaO2/FiO2	153.93	58.57	371.1	318.6	140.5	442.61	0.113

*Statistical significance was obtained with the non-parametric Mann–Whitney U test.

### Relation between D-dimer, disseminated intravascular coagulation, and nutritional status with survival

**FIGURE 1:**
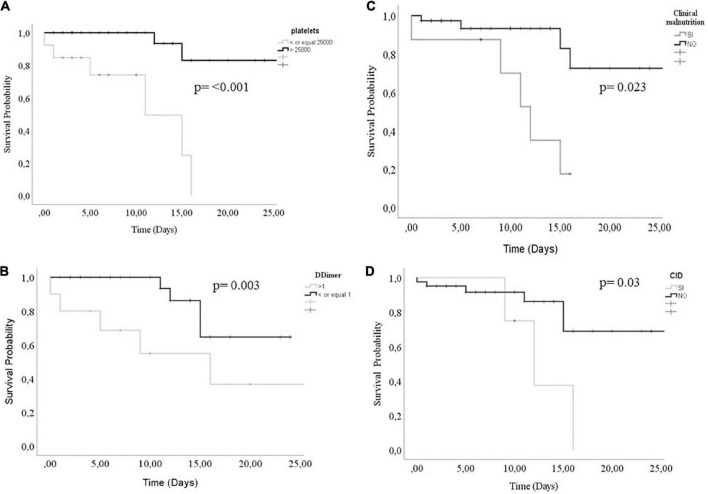
Survival curves comparison according to panel **(A)** platelet count, **(B)** D-dimer concentration, **(C)** Clinical malnutrition, and **(D)** CID status. A log-Rank value of <0.05 was considered statistically significant.

The log-Rank test was used to compare the Kaplan–Meyer curves for the survival assessment of the pediatric patients hospitalized for COVID-19; throughout 26 hospitalization days, the D-dimer, thrombocytopenia, disseminated intravascular coagulation (DIC), and clinical malnutrition status were associated with lower survival (log-Rank test, all *p* < 0.05 Figures 1A–D). The survival time median for a thrombocytopenia ≤ 25,000 was 11 days (Q_25_5-Q_75_15) *p* ≤ 0.001, Figure 1A. For a D-dimer concentration ≤ 1 ug/mL, the survival time median was 16 days (Q_25_5-Q_75_26) *p* = 0.003, Figure 1B. Regarding patients with low clinical malnutrition status, the survival time median was 12 days (Q_25_9-Q_75_15) *p* = 0.023 Figure 1C; the DIC was analyzed too, and a survival time median of 12 days (Q_25_9-Q_75_16) *p* = 0.03 was found in patients who had it, Figure 1D.

## Discussion

Mortality-associated factors in cancer and COVID-19 pediatric patients lack enough study; this is the first work to describe the factors associated with mortality and survival in Mexican patients with cancer and COVID-19; therefore, the available information regarding laboratory markers is still scarce.

Coronavirus disease 2019 disease in healthy children usually exhibits a favorable course and prognosis.

However, this topic has not yet been explored regarding pediatric cancer patients. Fever was the most frequent symptom found in this work which is similar to what has been previously reported in healthy adults (5) and children, followed by a cough that matches previous reports in adults (6, 7).

The mortality-associated factors for this cancer and COVID-19 pediatric patients’ cohort include stage IV tumor, intubation, saturation percentage, RDW, thrombocytopenia, creatinine and alanine aminotransferase (ALT), D-dimer, ferritin, and FiO_2_ percentage.

In Mexico, cancer mortality in children is (20.0%) (7); these patients are known to have a more critical illness from COVID-19, and mortality is even higher in children without a cancer diagnosis (8). It seems that the type of tumor plays an essential role in developing complications in the pediatric population, from 47 to 60% of the reported cases corresponding to leukemia and lymphoma. The reason attributed to an increased risk of complications from COVID-19 is because hematopoietic malignancies experience prolonged neutropenia and immunosuppression, and these children may be more susceptible to COVID-19 infection. Mukkada S et al. noted that an association of severe illness in low- and lower-middle-income countries might be related to differences in supportive care infrastructure and delays in patient approaches. In addition, health care system disruptions that affect all aspects of care delivery may be more pronounced in low- and lower-middle-income countries (9). Another reason for the high frequency of respiratory failure and high mortality observed in this study was that pediatric cancer patients did not go to their referral center for therapeutic follow-up due to families’ fear of exposing their children and themselves to the virus, which resulted in worse chances of survival due to loss of follow-up in their chemotherapies. This problem was observed not only in Mexico but also in other countries around the world. Other authors have shown changes in the care of these cancer patients, such as reduced surgical care, shortage of blood products, delays in imaging studies, modifications of chemotherapy, interruptions of radiotherapy, and abandonment of treatment (10–12).

The mortality-associated findings in adults with COVID-19 comprise D-dimer high levels and thrombocytopenia with a significant impact on coagulation; these results highlight the need for prognostic mortality markers for children.

Thrombocytopenia in cancer children is understudied, but a low count in adults was associated with coagulation disorders that lead to organ failure and death in patients with severe COVID-19 (13–18). In adult patients with hemato-oncological diseases and COVID-19, results were similar to the ones found in adults free of cancer (19, 20).

Thrombocytopenia was associated with mortality in this group of patients. We initially considered that this was probably a consequence of the active administration of chemotherapy that most patients in this series received before SARS-CoV-2 infection; however, this was ruled out because when analyzing whether chemotherapy was associated with an increased risk of mortality, no statistical significance was found (data not shown), as reported by a previous study, but adults (21). Some cohort studies evaluating patients with cancer (solid tumors) and COVID-19 found no significant risk of mortality from recent chemotherapy (22, 23). Another cohort study (*n* = 309 patients) reported that cytotoxic chemotherapy was not significantly associated with severe or critical illness from COVID-19 (HR, 1.10; 95% CI, 0.73 to 1.60); however, the type of cancer was associated with greater severity due to COVID-19. In addition, in this same study, lymphopenia was observed during the diagnosis of COVID-19, and this was associated with a severe or critical illness (HR, 2, 10, 95% CI, 1.50 to 3.10). Unfortunately, in this study, the thrombocytopenia and other laboratory parameters could not be determined before the diagnosis of COVID-19 (24). However, there is controversy with a previous report in which it was observed that the severity of COVID-19 is more significant in patients with hematological neoplasms and recent chemotherapy (administered in the last 4 weeks) (25).

Most reports have found elevated D-dimer levels associated with mortality; for example, D-dimer concentration above 2.0 μg/mL is associated with mortality (HR: 51.5, 95% CI: 12.9–206.7) (26) in healthy adults with COVID-19. In a systematic review study, M Sakka et al. (27) found high D-dimer levels in COVID-19 deceased patients [3.59 μg/L (95% CI 2.79–4.40 μg/L)]; D-dimer value over 4.6 μg/mL [95% CI, 2.8–6.4; *p* < 0.00001] was associated with mortality in COVID-19 hospitalized patients (28). Another study reported that D-dimer levels ≥ 1.0 μg/ml at admission were associated with increased hospital mortality (29). In general, the studies carried out so far in adults with COVID-19 have revealed that increased D-dimer levels suggest an extensive thrombin production and fibrinolysis, thus developing a poor prognosis (30–33).

Disseminated intravascular coagulation is a coagulopathy characterized by thrombocytopenia, elevated D-dimer levels, prolonged prothrombin time, and reduced fibrinogen; it has been associated with a poor prognosis in COVID-19 patients (34, 35). Results for this cohort resemble previous reports of the adult population with COVID-19.

Ferritin was another altered marker in this cancer and COVID-19 pediatric patients’ cohort. Literature shows that COVID-19 adults with high levels of ferritin (2998, *p* = 0.016) suffered respiratory failure and needed invasive mechanical ventilation, and also had a greater mortality risk (36). Lino et al. (37) found elevated mortality OR 6.0 [95% CI = 1.4–26.2; *p* = 0.016)] in adults sickened by COVID-19 with high ferritin levels ≥ 1873.0 ng/mL.

Another study conducted by Deng et al. (38) in COVID-19 patients from the intensive care unit showed that the group with elevated ferritin levels was associated with higher mortality, OR 104.97 [95% CI = 2.63–4185.89; *p* = 0.013].

Regarding the creatinine medians between living and dead, statistically significant differences were found, although the medians of both groups do not indicate that these levels are out of range (0.28 vs. 0.59, respectively). However, at the 75th percentile, only in the deceased is the number of standard values exceeded.

Interestingly, thrombocytopenia, D-dimer, nutritional status, and disseminated intravascular coagulation were significantly associated with reduced survival in this cohort.

The literature proposes two hypotheses regarding thrombocytopenia in COVID-19 patients. The first one proposes coronaviruses *per se* lead to a hematopoietic inhibition; and the second one proposes the lung as one of the organs where mature megakaryocytes release thrombocytes; hence, the thrombocytopenia may be associated with lung damage in COVID-19 patients (39, 40). Nevertheless, the specific pathway behind thrombocytopenia is poorly studied.

The mechanism by which D-dimer increases in COVID-19 patients is not fully understood either. However, it is proposed that the observed elevation implies a hyperfibrinolysis status and an inflammatory load increase triggered by the SARS-CoV-2 infection (41, 42).

Several mechanisms contributing to the coagulation disorder observed in the DIC have been proposed. The coagulation onset and propagation, simultaneous impairment of anticoagulation pathways, and an endogenous fibrinolysis deficit, all as a systemic inflammatory activation outcome, give rise to platelet activation and fibrin storage. Eventually, these lead to platelets, and procoagulant factors intake, thus causing an associated bleeding diathesis (43).

## Conclusion

Fever was the most frequent symptom; acute respiratory failure was the main complication, followed by added infections; the clinical parameters associated with mortality were stage IV tumor, intubation, saturation percentage, RDW, thrombocytopenia, creatinine, ALT, D-dimer, ferritin, and FiO_2_ percentage. The thrombocytopenia, D-dimer, nutritional status, and disseminated intravascular coagulation were significantly associated with lower survival.

## Limitations

This is a retrospective study with a limited sample size in a single institution; however, one advantage of it is having a captive population (cohort), which has defined and validated variables (clinical laboratories, comorbidities, treatment, and survival).

## Data availability statement

The raw data supporting the conclusions of this article will be made available by the authors, without undue reservation.

## Author contributions

L-MH-R, LT-E, and MD-L performed the search strategy and selection criteria and wrote and reviewed the manuscript. LT-E, MD-L, and AA-G wrote and reviewed the manuscript. LV-H, AG-G, MA-O, and RC-C provided the clinical data, nutritional status data, and reviewed the manuscript. All authors contributed to the article and approved the submitted version.
